# A double-blind, randomized controlled, prospective trial assessing the effectiveness of oral corticoids in the treatment of symptomatic lumbar canal stenosis

**DOI:** 10.1186/1477-5751-13-13

**Published:** 2014-08-07

**Authors:** Luiz Claudio L Rodrigues, Jamil Natour

**Affiliations:** 1 Orthopaedic Department, Santa Marcelina Hospital, São Paulo, Brazil; 2 Disciplina de Reumatologia, Universidade Federal de São Paulo – UNIFESP, Rua Botucatu 740, Sao Paulo, SP CEP: 04023-900, Brazil

**Keywords:** Spine, Lumbar stenosis, Treatment, Neurogenic claudication, Corticoid

## Abstract

**Background:**

Corticoids have potent anti-inflammatory effects, which may help in relieving pain and dysfunction associated with lumbar canal stenosis. We assessed the effectiveness of a decreasing-dose regimen of oral corticoids in the treatment of lumbar canal stenosis in a prospective, double-blind, randomized, placebo-controlled trial.

**Results:**

Sixty-one patients with lumbar canal stenosis (50–75 years; canal area < 100 mm^2^ at L3/L4, L4/L5, and/or L5/S1on magnetic resonance imaging; and claudication within 100 m were electronically randomized to an oral corticoid group (n = 31) or a placebo group (n = 30). The treatment group received 1 mg/kg of oral corticoids daily, with a dose reduction of one-third per week for 3 weeks. Patients and controls were assessed by the Short Form 36 Health Survey, Roland–Morris Questionnaire, 6-min walk test, visual analog scale, and a Likert scale. All instruments showed similar outcomes for the corticoid and placebo groups (P > 0.05). Obese patients exhibited more severe symptoms compared with non-obese patients. L4/L5 stenosis was associated with more severe symptoms compared with stenosis at other levels.

**Conclusion:**

The oral corticoid regimen used in this study was not effective in the treatment of lumbar canal stenosis.

## Background

Symptomatic lumbar canal stenosis is characterized by degenerative alterations in several structures of the vertebral segment, including the zygapophyseal joint, flavum, articular capsule, and intervertebral disc. These alterations decrease the vertebral canal area, resulting in pressure on the neural structures [1] that is clinically manifested as low back pain [2] and lower limb pain that worsens while walking and improves with rest, a presentation called neurogenic claudication [3].

As the population ages, the number of symptomatic patients is expected to increase. Indeed, symptomatic lumbar canal stenosis is the primary reason for surgical treatment of the spine in patients over 60 years of age [4]. Treatment of spinal stenosis begins with guidance about the disease, adequate pain control, physical therapy, and exercise [5] to maintain activities of daily living. If these measures fail, surgery may be necessary, particularly in patients with exercise intolerance, walking difficulty, and/or urinary incontinence [6]. While more than 50% patients respond satisfactorily to drug treatment and physiotherapy [7], those with severe stenosis and major neurological involvement may require surgery [8].

Because spinal stenosis most frequently affects the elderly, a patient group with a high surgical complication rate, an oral regimen can dramatically improve overall treatment safety. To this end, we assessed the efficacy of oral corticoids for the treatment of lumbar canal stenosis.

### Patients and methods

This prospective, double-blind, randomized controlled study was approved by the research ethics committee of Universidade Federal de Sao Paulo (CEP 1892/10). Sixty-one patients were electronically randomized to the drug treatment or placebo control group. Opaque envelopes were used to ensure the secrecy of the allocation. The drug treatment group was administered corticoids at 1 mg/kg/day with a one-third dose reduction per week. The control group was administered placebo for the same period. All patients were assessed at four time points during the study: baseline (T0), at the end of treatment (week 3, T3), and at 6 and 12 weeks after study initiation (T6 and T12, respectively). All patients were permitted paracetamol (750-mg tablets) up to 3 times a day as a rescue analgesic. Total paracetamol intake and response were also assessed. The patient and the assessor were blinded to treatment.

All patients were assessed by the following instruments: canal area calculation; a 10-cm visual analog scale (VAS) for pain; the Roland–Morris Questionnaire for function [9]; the 6-min walk test for function; the Short Form (SF)-36 Health Survey for quality of life; paracetamol consumption during the study and a 5-point Likert-type scale to evaluate the patient satisfaction with the treatment where the following question was done to patient: “Thinking in how you are before treatment, how do you fell now?” and they can choose between 5 options (“much better”, “slightly better”, “unchanged”, “slightly worse”, and “much worse”) – the Likert scale was not applied for the initial assessment (T0).

Inclusion criteria were the presence of claudication within less than 100 m and the presence of at least two of the following lower limb symptoms: pain, weakness, burning, tingling (associated with or independent of lower back pain), and a vertebral canal area of <100 mm^2^ in at least one of the levels assessed (L3/L4, L4/L5, L5/S1) by the Hamanishi technique [10].

The exclusion criteria were as follows: decompensated diabetes mellitus, systemic hypertension, decompensated cardiopathy, systemic diseases affecting the lower limbs, neuromuscular diseases, use of corticoids in the past 3 months, previous lumbar or thoracic surgeries, cognitive deficits that compromise the capacity to understand or interpret the questionnaires, spondylolisthesis (except degenerative), degenerative scoliosis with a Cobb angle of >10°, degenerative pathologies in the hip or knee that can interfere with gait, and a history of total or partial arthroplasty in the hip and/or knee joint.

The canal area was calculated on the basis of maximum anteroposterior (a) and mediolateral (b) area using magnetic resonance imaging (MRI). After calculating these values, they were individually divided by 2 and multiplied by π as show in the following formula:

$$\mathrm{Area}=\left(a/2\right)\times \left(b/2\right)\times 3.14\times \mathrm{constant}$$

The constant is 0.8 when the canal is circular, 0.7 when the canal is elliptical, 0.6 in the presence of facetary compression, and 0.5 when the compression is caused by the disc and facets [10].

The sample size was chosen to yield a statistical power of 80% and a α of 5% when comparing VAS scores between groups, assuming a group standard deviation of 2 cm and a minimum mean intergroup difference of 2 cm. We used Student’s t-test to compare continuous variables with a homogenous distribution across groups. Generalized linear models and unbalanced 2-factor ANOVA were used to test the temporal effect of medication and interaction between the test period and medication. Tukey’s test was used for 2 by 2 comparisons between time period and medication for the measurement points. Pearson’s correlation analyses were performed to assess the relationships among clinical variables. In all statistical tests, P-values of <5% were considered statistically significant.

## Results

All 61 eligible patients, including 31 in the corticoid group and 30 in the placebo group, completed the study. There were no group differences in female: male ratio and there were no significant differences in height and weight between groups for the males and females. The mean and standard deviation for age was 58.23 (6.38) in the corticoid group and 58.33 (6.19) in the control group. Regarding BMI the mean and standard deviation for the corticoid group and control group was 25.97 (5.16) and 27.90 (4.53) respectively, and no difference were found between groups.

The mean and standard deviation of the stenosis at the three levels assessed, namely L3/L4, L4/L5, and L5/S1, are presented in Table 1.

**Table 1:** Lumbar canal area

	**L3/L4**	**L4/L5**	**L5/S1**
**Mean**	103	84	84
**Standard deviation**	25.31	15.36	15.79

The VAS scores for pain suggested a mild improvement in both groups at T3, as indicated by the lower scores compared with those at baseline (T0); however, these scores increased thereafter, with no significant change across test periods between groups (Table 2).

**Table 2:** VAS score, Roland–Morris score and 6-min walk test distance before and after corticoid treatment

	**T0**	**T3**	**T6**	**T12**
	**VAS score (cm) P-value 0.56**			
**CORTICOID**	7.68	5.68	6.71	6.61
**PLACEBO**	7.07	5.50	5.17	5.97
**P-VALUE**	0.30	0.81	0.02*	0.37
	**Roland–Morris score P-value 0.40**			
**CORTICOID**	16.16	12.77	14.71	14.81
**PLACEBO**	15.27	14.73	13.80	13.80
**P-VALUE**	0.46	0.25	0.53	0.52
	**6-minute walk test (m) P-value 0.73**			
**CORTICOID**	367.39	379.97	346.8	352.6
**PLACEBO**	388.83	391.50	389.4	395
**P-VALUE**	0.36	0.62	0.07	0.08

VAS: visual analog scale; *p value statistically significant.

The Roland–Morris questionnaire was administered at baseline (T0), after the 3-week drug/placebo trial (T3), and at 6 and 12 weeks after study initiation (T6, T12) (Table 2). The scores suggested a slight improvement in the corticoid group at the beginning of treatment; however, they were not significantly different from those for the placebo group. In fact, no significant differences in total scores were observed within or between groups for any individual assessment period.

The 6-min walk test, performed according to the American Thoracic Society standards on a 22-m track with patients walking as fast as possible, also showed no significant improvement in both groups. In fact, the total distance travelled by the corticoid group decreased by approximately 40–50 m between T3 and T6 and T12 (Table 2). Because this assessment depends on muscular structure, the male and female subgroups were separately compared; this analysis also indicated no benefit of corticoids (not shown).

The SF-36 questionnaire assesses current health conditions, with higher values corresponding to a better condition. The SF-36 scores for our patients suggested small differences in some of the eight domains during the study period; however, the final values at T6 and T12 were similar between groups (Table 3). Separate comparisons by gender also did not reveal significant differences in condition between groups (not shown).

**Table 3:** Short form 36 domain scores before and after corticoid treatment

	**T0**		**T3**		**T6**		**T12**		
	**Corticoid**	**Placebo**	**Corticoid**	**Placebo**	**Corticoid**	**Placebo**	**Corticoid**	**Placebo**	**P value**
**FUNCTIONAL CAPACITY**	33.55	26.27	42.35	38.17	38.71	43.17	37.26	41.67	0.49
**PHYSICAL LIMITATION**	19.35	17.50	44.52	48.83	18.55	31.67	20.16	26.27	0.74
**PAIN**	28.06	31.60	46.39	43.70	36.48	44.20	43.42	42.00	0.55
**GENERAL HEALTH STATUS**	45.00	39.63	47.65	45.20	45.94	46.93	45.87	45.07	0.85
**VITALITY**	45.06	45.33	51.77	51.13	46.94	46.00	47.90	45.23	0.99
**SOCIAL ASPECTS**	46.68	56.58	60.58	68.37	65.73	55.25	53.74	52.42	0.21
**EMOTIONAL ASPECTS**	35.47	34.67	50.54	59.30	60.21	65.56	56.13	53.33	0.90
**MENTAL HEALTH**	49.81	49.73	55.74	55.47	55.48	59.00	55.23	52.93	0.93

Regarding the paracetamol use as a rescue analgesic between groups over the 21-day drug/placebo administration period, we found that the corticoid group intake was 19.42 (11.79) and the control group intake was 19.63 (11.29) and no significant difference was observed between groups (p = 0.94).

The Likert-type scale, wherein patients were instructed to document how they were feeling after treatment (much better, slightly better, unchanged, slightly worse, or much worse) also indicated no significant benefits of corticoids.

The body mass index (BMI) show a low correlation with VAS for pain, (r = 0,34 and p = 0.035) Roland Morris scores (r = 0,27 and p = 0.041), six minute walk test (r = 0,31 and p = 0.039) and SF-36 (r = 0,22 and p = 0.031). Showing that increase in BMI did not lead to more pain or poor function and quality of life.

Regarding the canal area we found a low correlation between lumbar canal stenosis at L3/L4 and more paracetamol consumption (r = -0,30 and p =0,016) and pain subscale of SF-36 (r = 0,25 and p = 0.049) showing that smaller canal area at L3/L4 leads to pain increase and makes patient’s to consume more analgesic medication.

Stenosis at L4/L5 shows a low correlation with VAS for pain (r = 0.28 and p = 0.027), showing that patients with stenosis at L4/L5 level have worse pain.

## Discussion

The oral corticoid regimen tested in this study provided no significant pain relief or benefits to motor function in patients with lumbar canal stenosis. The primary structures contributing to the decrease in spinal canal area and many of the symptoms of spinal stenosis are the intervertebral disc, zygapophyseal joint, flavum, and venous plexus [10]. However, not all patients that present with signs of stenosis on spinal MRI also present with clinical symptoms [11]. Inflammation of these structures will also compress the cauda equina roots within the vertebral canal and as they pass through neural foramen [12]. In this study, the patients presented with diffuse symptoms in lower limbs and it was not possible to define a myotome for each patient. However, Schonstrom and colleagues concluded that the hallux extensor is the most severely affected muscle in patients with symptomatic lumbar canal stenosis [11]. This study assessed the movement of joints that could mimic claudication due to stenosis by altering function at the hip or knee [12]; however, patients with symptomatic alterations that could influence gait were excluded from the study. The present study also did not include patients with fibromyalgia or depression, comorbidities that can amplify symptoms [13].

In this study, the area of stenosis did not influence evolution of the disease, and similar to the findings of Zeifang, we found no relationship between the distance walked and canal area. Also, in accord with our study, Thornes et al. [14] found that gender did not influence the clinical response. In the present study, excess weight worsened prognosis and directly influenced patient performance in the 6-min walk test, a result not consistent across studies. Crosgrove et al. [15] reported that BMI was not predictive of treatment response. As in the current study, Briggs et al. [16] reported that patients with a high BMI reported more severe pain, but these patients did show clinical improvement after epidural corticoid treatment. Many of the clinical variables examined in the current study, such as gender, BMI, canal diameter, and walking capacity, have also been examined previously. In the current study, canal diameter neither influenced clinical status nor predicted therapeutic response. On the basis of canal diameter assessments, Campbell et al. [17] tried to identify patients who would best respond to epidural corticoids, obviating the need for surgical treatment. Although they used a different intervention, all patients used epidural corticoids, and the results showed that canal diameter was not predictive of treatment response. In accord with our study, patients with L4/L5 stenosis were more symptomatic, albeit with no statistically significant differences in disease evolution.

Our study included cases of degenerative spondylolisthesis, all between L4/L5. This eventually causes stenosis of the cauda equina roots [15,16]. In the present study, all patients with spondylolisthesis were female, Sengupta et al [18] concluded that there was little difference in efficacy among treatments, regardless of the degree of slippage.

This study attempted to identify an effective oral medication regimen for the management of spinal stenosis, while maintaining safety by using a decreasing-dose regimen. While this treatment appeared safe, with no obvious adverse events, it was not effective for pain or movement deficits. The effectiveness of corticoids in providing relief from this syndrome has been tested several times, most often when applied locally, and the clinical response has been highly variable [17,19-26]. Campbell et al. [17] reported that canal diameter is not predictive of response to epidural steroid injection in patients with lumbar canal stenosis. Ng et al. [27] reported no effect of corticoids in a study of patients with lumbar canal stenosis that compared anesthetics plus epidural corticoids with anesthetics alone. While these results are in general agreement with ours, Sayegh et al. [28] report clinical improvement with epidural corticoids. The reasons for these discrepancies remain to be explored, although it should be noted that our assessment instruments were the same as those used in many of the aforementioned studies, and all have been validated for the study of stenosis symptomology and relief [28-40].

Obesity, often due to a sedentary lifestyle, is associated with loss of sagittal balance, changes in muscle strength, and a greater weight load on vertebral discs, all of which are likely to exacerbate degeneration. These patients are more susceptible to painful conditions and generally carry a poorer prognosis for most diseases. In this study as well, we found that patients with a higher BMI were more symptomatic, suggesting that weight loss is one safe noninvasive therapy that may improve symptoms and possibly obviate the need for surgery.

The structures compression in lumbar canal stenosis leads to two changes: one caused by mechanical compression and another by venous dilatation and the venous blood retention can lead to local vascular inflammation and subsequent pain. These changes can cause neuropathic pain that can recruit inflammatory mediators enhancing the inflammation component. Probably the mechanical compression and edema during gait are the main causes of pain and inflammatory component is only a secondary adjunct in this disease [41]. We assume it, although we cannot prove that the inflammatory component is the least important.

Studies showing no effect of these potent anti-inflammatory drugs on stenosis symptoms suggest that inflammation is not important for symptom expression and that mechanical and anatomical alterations are more likely explanations. However, we do not know how effective this particular dose regimen was at decreasing inflammation around the stenosis sites; therefore, we cannot exclude a beneficial effect of other more efficacious and tolerable anti-inflammatory drugs.

## Conclusions

This placebo-controlled study indicates that a tapering regimen of oral corticoids, starting at 1 mg/kg daily, is not effective regarding pain, function, quality of life, analgesic consumption or satisfaction with the treatment in patients with lumbar canal stenosis when compared to placebo. Obese patients with lumbar canal stenosis show more pain or poor function and quality of life. Lumbar canal stenosis at L3/L4 leads with more analgesic consumption as compared with stenosis in other levels and lumbar canal stenosis at L4/L5 is more painful as compared with stenosis in other levels.

### Key points

• The use of oral corticoids is not effective in the treatment of lumbar canal stenosis.

• Obese patients with lumbar canal stenosis show more pain or poor function and quality of life.

• Lumbar canal stenosis at L3/L4 leads with more analgesic consumption as compared with stenosis in other levels.

• Lumbar canal stenosis at L4/L5 is more painful as compared with stenosis in other levels.

## Competing interests

The authors declare that they have no competing interests.

## Authors’ contributions

LCLR held the inclusion of patients, study design, statistical evaluation and formatting article. JN worked in study design, participated in its coordination and helped to draft the manuscript. All authors read and approved the article.
